# A Case of New-Onset Obsessive-Compulsive Disorder and Schizophrenia in a 14-Year-Old Male following the COVID-19 Pandemic

**DOI:** 10.1155/2023/1789546

**Published:** 2023-06-10

**Authors:** Daniel Bibawy, Jennyferd Barco, Yeghia Sounboolian, Priya Atodaria

**Affiliations:** ^1^Richmond University Medical Center, Staten Island, USA; ^2^St. George's University, St. George's, Grenada

## Abstract

The post-COVID-19 era has introduced novel cases of psychiatric complications that are either organic or purely psychological in nature due to social isolation. This report details a case of new-onset obsessive-compulsive disorder (OCD) and schizophrenia following the COVID-19 pandemic. The novelty of this case lies in the onset of the patient's symptoms in the context of the COVID-19 pandemic, without prior predisposing risks in the environmental, social, or biological aspects. We provided therapeutic treatment to the patient in an inpatient setting, while examining the patient to decipher the root cause of his symptoms. While there is substantial data suggesting exacerbations of OCD during the COVID-19 pandemic in the general population and a new onset of schizophrenia due to the virus itself, very little is known about the prevalence of either OCD or schizophrenia after the pandemic. With this in mind, we hope to provide more information regarding new-onset psychosis and OCD within the adolescent population. A considerable amount of studies and data are needed in this subset of the population.

## 1. Introduction

The coronavirus disease 2019 (COVID-19) pandemic changed much of how individuals interact as social beings. During its peak, the COVID-19 pandemic led to widespread social isolation. Most preteens and early teens were subject to this isolation during a crucial time in their social maturation [1]. Though older adults with comorbidities are more susceptible to developing serious physical complications of COVID-19, children and adolescents have had to endure biopsychosocial stressors brought on by the pandemic as their daily routines were unexpectedly disrupted [2, 3].

Studies have indicated that isolation and prevention strategies, such as quarantining, have led to depression, anxiety, mood disorders, sleep disorders, and posttraumatic stress symptoms [4]. Similar isolative measures put in place during the COVID-19 pandemic have been shown to be detrimental to mental health [5]. In addition, research has linked the biopsychosocial stressors brought on by COVID-19 to depression, anxiety, helplessness, worry, fear, inattention, clinging, worry, and irritability [5–7]. In the general adult population, studies found an increase in mental health illnesses during the pandemic, with younger and financially insecure adults at a higher risk [8–10]. Additional risk factors included living alone, having preexisting mental and physical health conditions, living in deprived areas, being an ethnic minority, and being of the female gender [11].

In addition to the depressive and anxiety-related symptoms brought on by the pandemic, the virus itself has also been linked to rare neuropsychiatric complications [12]. Furthermore, multiple case reports have alluded to the emergence of new-onset obsessive-compulsive disorder in both adolescents and adults, along with exacerbations of preexisting OCD during the COVID-19 pandemic [13–16]. However, only a small subset of case reports has linked COVID-19 infection with psychosis in adults as well as adolescents [17, 18]. Though there is a growing literature of new-onset OCD during the pandemic, the association between the pandemic and new-onset psychosis is still poorly understood. The incidence of childhood-onset schizophrenia is less than 0.04%, and there is inadequate information regarding new-onset childhood schizophrenia following the COVID-19 pandemic in adolescents.

This case report presents a 14-year-old adolescent male with new-onset obsessive-compulsive disorder and schizophrenia following the COVID-19 pandemic to provide insight and offer information to the literature, assist with future management, and guide additional research.

## 2. Hospital Course

The patient is a 14-year-old Caucasian male, domiciled at home with his parents, 21-year-old brother, and 19-year-old sister, attending 9th grade in regular classes with para, with no known past medical history and a past psychiatric history of attention-deficit/hyperactivity disorder (ADHD) which had been recently diagnosed by his neurologist, who was brought by his parents to the psychiatric emergency room for evaluation of bizarre behavior.

During the patient's walk toward the evaluation room, he was noted to be talking to himself. Upon evaluation, the patient was in no acute distress, awake, alert, and oriented to self, place, and time, appropriately groomed, calm, and evasive with the evaluation. The patient appeared anxious and sat with his arms crossed throughout the evaluation. His affect was flat. The patient was minimally verbal throughout the evaluation, often trying to answer questions with thumbs up or down. He had a concrete thought process and exhibited poverty of thought and thought blocking, often taking several minutes before responding to a question. When asked about his favorite subject in school, he stated “anything” and had the same response for hobbies, interests, and plans for the future. When asked about the reason for the patient's presentation, he stated “For safety.” When pushed further, he stated “Because I have” and then stopped talking and refused to answer despite encouragement. The patient reported he sleeps roughly 7 hours per night, and when asked for the time, he stated from 8 pm to 6 am, and when asked to calculate the number of hours again, he stated “13,” indicating difficulty with attention and simple mathematics at a level below expected for his age. The patient denied any emotional, verbal, physical, or sexual abuse. He denied auditory and visual hallucinations although at times appeared to be responding to internal stimuli, speaking to himself. The patient denied suicidal ideation, homicidal ideation, intent, or plan. He denied using nicotine, alcohol, marijuana, and illicit drugs.

A collateral history was obtained from the patient's mother, who accompanied the patient to the hospital. She stated that the patient hit all developmental milestones on time with no difficulty. The reported patient used to be “a normal, kinda quiet” child. She reported that he was attending a charter school with no complaints, and when the COVID-19 pandemic started, he had to do school remotely from home. During that time, his mother noticed that the patient had become “germaphobic,” washing repeatedly and refusing to leave without a mask despite encouragement when restrictions were being lifted. She reported that he would repeatedly wash his hands multiple times throughout the day, stating that he would spend close to one hour daily washing his hands. He returned to his original school after COVID-19 and was noted to be more shy, less social, and less interactive than he had been before the pandemic. The patient then transitioned into another school and was noted to continue behaving differently than he had prior to the pandemic. His mother reported that the patient would only stand in a corner, adamantly refusing to engage and avoiding crowds. She reported that the school offered an individualized education program (IEP) and a para; the IEP was refused, and the para was accepted despite the patient's resistance. The mother reported that in September of 2022, the patient lost a relative whom the patient was very close with very unexpectedly, unrelated to the COVID-19 pandemic. She reported that since then, the patient had been declining in functioning. He had become almost nonverbal even with his long-time friends. He was noted to get up and dance in the corner of the classroom in the middle of class last week. The patient had also started self-urinating. His mother first noticed small stains on his underwear, but one week before admission when riding the subway, the patient fully urinated on himself without appearing to feel bothered or embarrassed. She also reported that the patient had stopped showering. He had started having “counting tics,” constantly counting under his breath. She reported that he continued to have fair sleep and appetite throughout this time. The mother stated that the patient had been recently evaluated by a neurologist who did an electroencephalogram (EEG) which resulted as normal and was then diagnosed with ADHD and prescribed Vyvanse 20 mg PO daily, which she had not been giving to the patient as she did not agree with the diagnosis of ADHD. She denied the patient using any drugs or alcohol, to the best of her knowledge.

The patient was hospitalized for 7 weeks in an acute child and adolescent psychiatric unit due to disorganized behavior and an inability to participate in activities of daily living (ADLs). Routine vitals and labs were taken throughout the hospitalization. Head CT was taken on admission to rule out underlying organic causes of the patient's behavior, and no acute or chronic abnormalities were noted. Head MRI was subsequently taken and resulted in an essentially normal MRI of the head, with a small pineal cyst (0.6 cm) of no clinical significance. The patient was found to have a reactive Lyme IgG 41 kDa band with no clinical significance. The patient was in no apparent distress throughout the hospitalization. Based on the patient's disorganized behavior such as urinating on himself and dancing in the middle of class and negative symptomatology, observed by flattened affect and alogia, leading to a disturbance in his ability to function in self-care as well as in the school environment for a period of greater than 6 months, the patient was found to meet DSM-5 criteria for schizophrenia. The patient also met the criteria for OCD, based on his compulsions which consisted of frequently washing hands for roughly one hour each day, as well as avoiding touching doorknobs in response to his fear of germs following the COVID-19 pandemic, with poor insight into his symptoms. The patient at times throughout the hospitalization would acknowledge that he was afraid to touch doorknobs due to fear of the spread of germs related to the COVID-19 pandemic; at other times, he refused to acknowledge or respond to questions related to compulsive hand washing and avoidance of doorknobs. The patient was given a dual diagnosis of OCD and schizophrenia. The patient was placed in a supportive structure, milieu, and individualized as well as group therapy. The patient was started on risperidone which was initially started at 0.5 mg at bedtime for disorganized behavior, lorazepam 0.5 mg PO daily for anxiety, and sertraline which was started at 25 mg PO daily and increased after 7 days to 50 mg PO daily for OCD. The patient showed improvement without side effects to treatment. At first, the patient continued to maintain bizarre, disorganized behaviors, such as pacing the same 4 steps in the hallway throughout the day, refusing to open or close doors, dancing in the middle of class, and urinating on himself without appearing to feel distressed or uncomfortable. As a result, medications were titrated, namely, risperidone, which was increased to 0.5 mg PO BID after 7 days; however, these behaviors continued at this dosage. Risperidone was again increased to 1 mg PO BID after another 5 days, and the patient appeared to respond to this dose of medication. As time progressed, with titration of medication and continued structure and therapy, disorganized behaviors such as dancing in the middle of class, repetitive pacing, and self-urinating ceased. However, the patient was never able to provide an explanation for why these behaviors took place aside from “I wanted to” or why these behaviors ceased (“I don't want to anymore”).

At the time of discharge, the patient's thought process remained largely concrete. He continued to respond to questions matter-of-factly and showed difficulty with abstract thinking, or providing reasons for his actions. However, his behavior had improved. He was calm, cooperative, and more organized in his thoughts and behavior. Psychomotor activity was within normal limits. His speech was hesitant and low in rate, rhythm, and volume, but spontaneous, compared to admission when speech was nonspontaneous. The patient interacted with peers and staff appropriately. Sleep and appetite were fair. The patient felt comfortable opening and closing doors by himself, compared to admission when he would not touch any door handles for fear of germs. The patient was future-oriented at the time of discharge and looking forward to spending time with his family and friends in school. The patient was able to care for his ADLs in an age-appropriate fashion. He denied current or past suicidal intent/homicidal intent/auditory and visual hallucinations. Impulse control was good. Insight and judgment were fair. At the time of discharge, his parents did not believe that the patient continued to be a danger to himself or others, and they were comfortable at this time for the patient to be discharged back to his home and follow up with psychiatry as an outpatient. Discussions with parents throughout the hospitalization indicated that the patient had returned to baseline since the COVID-19 pandemic; however, they indicated that before the pandemic, the patient had shown no signs of psychiatric illness. Per his parents, the patient previously interacted with his peers appropriately, met all developmental milestones, and was doing well in all classes. They adamantly denied any family history of psychiatric illness and denied any substance use history in the patient to the best of their knowledge. The patient also denied any substance use history, and toxicology screens obtained in the hospital resulted as negative. As any underlying medical causes have at this point been ruled out, we can assert with reasonable confidence that this patient presented with a primary case of new-onset schizophrenia and new-onset OCD following the COVID-19 pandemic.

## 3. Discussion

The current case illustrates early-onset schizophrenia and obsessive-compulsive disorder in a patient with no prior psychiatric history or family history of psychiatric conditions. Schizophrenia can be divided into early-onset schizophrenia (EOS) that occurs in adolescents between the ages of 13 and 17 and very early-onset schizophrenia (VEOS) that presents at or before 12 years of age [19].

The patient met all developmental milestones accordingly prior to the initiation of the COVID-19 pandemic. At the start of the COVID-19 pandemic, the patient began to become more withdrawn and demonstrated repetitive behavior such as repetitive hand washing. The patient eventually refused to leave the house without a mask after the wave of the pandemic. The patient became nonverbal toward his family and friends but eventually began to have disorganized speech as represented by thought blocking and nonspontaneous “matter of fact” fashion and disorganized thought. The disorganized behavior began when the patient returned to school after the peak wave of the COVID-19 pandemic. He began to get up in the middle of the classroom and dance and eventually started urinating on himself and stopped showering. The patient was not able to participate in activities of daily living. Initial evaluation to rule out organic causes included EEG by the neurologist, head CT and MRI, and laboratory evaluation which included a drug analysis. The patient was originally diagnosed with ADHD by the neurologist, but with the progression and worsening of symptoms, the diagnosis was changed to obsessive-compulsive disorder and early-onset schizophrenia. Autism spectrum disorder was ruled out due to age of onset, degree of symptoms, and no prior history of developmental delay until this point. The patient was able to reach his baseline through the current medication regimen with scheduled outpatient follow-up. The patient began to vocalize more, and his disorganized speech improved slightly, although he was never able to reason his behaviors and continued to show difficulty with abstract thinking. The pandemic itself became a risk factor for either enhancing or developing psychiatric illnesses within the child and adolescent population, who are vulnerable to such drastic changes in their social environment.

Children and adolescents' mental health has been primarily affected through high exposure of biopsychosocial stressors generated by the COVID-19 pandemic [20]. The disruption of daily life routine and social isolation from school environment and peers, and with their inability to comprehend the consequences of the pandemic, have led many children and adolescents to experience stress [7]. In children and adolescents, stress may manifest as anxiety, irritability, insomnia, and clingy behavior related to emotional distress from experiencing social isolation and the death of relatives during the pandemic [21]. The patient experienced social isolation when he began remote learning. This was when the mother first began noticing signs and symptoms of obsessive-compulsive behavior. Throughout the hospital course, the patient's diagnosis was changed from ADHD to both OCD and early-onset schizophrenia. Previous research showed that obsessive-compulsive disorder is frequently observed in patients during their first episode of schizophrenia in young adults [21]. However, recent research showed that a substantial proportion of adolescent schizophrenia patients are also diagnosed with OCD [22]. The patient's profile supports the recent literature findings of being diagnosed with early-onset schizophrenia and OCD.

Within recent years, there have been case reports illustrating new-onset mania and psychosis in adolescents with asymptomatic COVID-19 infections [23]. The hypothesis in these cases is that the COVID-19 infection potentially triggered or exacerbated psychosis and mania in these patients [23]. There were a few confounding variables in these case reports which include prior psychiatric history and substance abuse disorder. It is unclear in those reports if new-onset mania and psychosis were triggered by COVID-19 infection or biopsychosocial stressors that the pandemic brought. Nevertheless, these patients were able to be stabilized and discharged on olanzapine. In the present case, the patient did not have a prior medical history of COVID-19 infection or a psychiatric history. There were minimal confounding variables that could have explained the patient's new-onset psychiatric symptoms. According to the patient's parents, the patient met all developmental milestones throughout childhood, and he did not begin to have symptoms until the pandemic. His symptoms began with repetitive handwashing and progressed to disorganized behavior and thought. The patient was able to be stabilized and discharged on risperidone, lorazepam, and sertraline regimen.

There have been similar reports of OCD and psychosis presentations in adolescence during the COVID-19 pandemic [13]. In that case report, the patient also had no prior psychiatric history but did have a positive urine drug sample for cannabis. The patient presented with similar obsessive-compulsive behavior such as repetitive cleaning of home and not wearing clothes outside of his bedroom, suicidal ideation, and auditory hallucinations [13]. The continued isolation, fear, and anxiety that the COVID-19 pandemic brought was a significant factor in that patient's worsening symptoms. This is similar in presentation to this current case report in which the biopsychosocial stressors of the pandemic played a role in the patient developing new-onset obsessive-compulsive behavior and disorganized behavior and thought. The difference is that the patient did not have a history of drug abuse or a positive urine drug screen. The patient also never expressed suicidal ideation. It is unclear if the patient ever experienced auditory hallucinations, since he would not answer questions in a straightforward manner and showed abstract thinking. Additionally, the patient's psychiatric symptoms continued to worsen even after he was able to attend school in person.

Studies have shown exacerbation of OCD in patients with a past psychiatric history by the COVID-19 pandemic with more than half of the patients having worsening symptoms during that time [24]. Yet, there is still limited research on how the COVID-19 pandemic has impacted the development of new-onset psychiatric illnesses in children and adolescents with no prior psychiatric or substance abuse history. This current case illustrates how the pandemic has possibly played a direct role in the development or enhancement of psychiatric illnesses in a 14-year-old adolescent male and has shown that this population is at risk for the development of new-onset psychiatric illnesses due to the stressors that the pandemic has brought.

## 4. Conclusion

The current case report highlights the advent of both OCD and schizophrenia in an adolescent in the period of the COVID-19 pandemic. With the social barriers and distancing decreasing and individuals slowly accommodating to the new postpandemic environment, we may see a potential rise in psychiatric evaluations. However, there is very little research and studies conducted in the evaluation of either psychosis or OCD in the post-COVID-19 era, especially in the child and adolescent population. Hence, screening protocols in the primary care setting should be increased. Though with our current case we are unsure if the psychosis was discovered earlier on or late in the patient's course at the time of the hospital admission, it was interesting to see how various psychosocial factors may have affected the patient's current presentation, eventually seeing the progression back to the patient's baseline through structured therapy and medications. As more cases, such as this, are discovered, we will be able to readily recognize and possibly prevent the course of such disorders from worsening. Through emphasizing the importance of screening initiatives within both the primary care and social settings, such as in schools, we hope to see an increase in the incidences of diagnosing psychiatric disorders within the aforementioned subtype of the population.

## 5. Limitations

Limitations to this case report include lack of tracking the severity and progression of symptoms at the start of the pandemic and tracking developmental milestones reached by the patient before symptom onset. Further limitation includes patient's reluctance in self-reporting all symptoms and therefore much of patient's symptomatology requiring observation with a limited history.
